# Physicochemical characterization and valorization potential of açaí berry (*Euterpe precatoria*) and bataua (*Oenocarpus bataua*) seeds: lignocellulosic biomass and antioxidants from Bolivian Amazon agroindustrial residues

**DOI:** 10.3389/fnut.2026.1835492

**Published:** 2026-06-04

**Authors:** Camila Rosales, Carol Mejía, Marcos Luján, Rosa Zabalaga, Daniel M. Larrea-Alcázar

**Affiliations:** 1 Centro de Investigación en Ciencias Exactas e Ingeniería (CICEI), Departamento de Ciencias Exactas e Ingeniería, Universidad Católica Boliviana “San Pablo”, Cochabamba, Bolivia; 2 Programa de Ciencia y Tecnología (PCT), Asociación Boliviana para la Investigación y Conservación de Ecosistemas Andino-Amazónicos (Conservación Amazónica-ACEAA), La Paz, Bolivia

**Keywords:** agroindustrial residues, Amazon, antioxidants, Bolivia, cellulose, circular economy, lignocellulosic biomass, Pando

## Abstract

Açaí berry (*Euterpe precatoria*) and bataua (*Oenocarpus bataua*) are palms whose fruits are harvested in the Bolivian Amazon for pulp production, generating large volumes of seed residues that are currently discarded without further processing. This study presents a comparative physicochemical characterization of both seed types, evaluating their proximate composition, structural fractions (cellulose, hemicellulose, lignin), dietary fiber, lipids, proteins, and antioxidant properties. Characterization was performed following internationally recognized methods (AOAC, ISO, NB). Results confirmed a predominantly lignocellulosic matrix in both species, with total dietary fiber exceeding 84% on a dry basis. Açaí seeds exhibited higher cellulose content (51.44 ± 0.56% dry basis) and considerable antioxidant activity (IC_50_ = 4.56 ± 0.11 mg/mL by DPPH; TEAC = 4,975.68 ± 56.21 μmol TE/g), açaí seeds exhibited a high total phenolic content (3,565.95 ± 21.78 mg GAE/100 g), while bataua seeds showed a balanced cellulose–hemicellulose ratio (32.58 ± 0.15% and 31.89 ± 0.59%, respectively), high total phenolic content (797.25 mg GAE/100 g), and moderate antioxidant capacity (TEAC = 1,567.10 ± 16.10 μmol TE/g). A multi-criteria valorization matrix identified cellulose and antioxidant compounds as the priority fractions in both species, supporting an integrated extraction cascade. Alkaline extraction experiments using NaOH demonstrated effective delignification and cellulose enrichment, with temperature identified as the dominant factor controlling cellulose yield in bataua seeds. FTIR spectroscopy and polarized-light microscopy confirmed cellulose structural integrity and crystallinity in both species. The integrated valorization of these residues—antioxidant extraction followed by cellulose recovery from the remaining solid—is proposed as a circular-economy strategy aligned with sustainable bioeconomy principles and Bolivian environmental legislation for the Amazonian region.

## 1 Introduction

The Bolivian Amazon, particularly the department of Pando, harbors exceptional biodiversity and represents a strategic territory for sustainable forest-based livelihoods (1). Among the non-timber forest resources of this region, açaí berry (*Euterpe precatoria* Mart.) and bataua or patauá (*Oenocarpus bataua* Mart.) are two Arecaceae palms whose fruits have become increasingly important for local communities (49). Açaí berry production in Pando has grown substantially, with the Reserva Nacional de Vida Silvestre Amazónica Manuripi (RNVSA Manuripi) reporting over 150 tonnes of açaí pulp processed in 2024 (2). Similarly, bataua represents a strategic income source with a regional production potential estimated at 1.2 million tonnes of fruit per year, though actual harvesting remains far below this potential (1, 3).

The extraction of pulp from both fruits generates substantial quantities of seed residues. In açaí, the seed represents approximately 85% of the total fruit weight (4), while in bataua, the seed accounts for 55–70% of the fruit (5). These residues are mostly discarded without treatment, creating environmental pressures in communities that lack adequate waste management infrastructure. From a resource perspective, however, both seed types constitute lignocellulosic biomasses that may harbor fractions of high industrial interest, including cellulose, hemicellulose, lignin, and bioactive compounds.

The circular bioeconomy framework promotes the transformation of agricultural and agroindustrial by-products into value-added materials, reducing waste generation while creating new economic opportunities (6). For Amazonian communities, the valorization of palm seed residues represents an opportunity to strengthen bioeconomy initiatives without requiring additional land use or deforestation. Açaí berry seeds have received growing scientific attention as sources of cellulose (7), antioxidant phenolics (8, 9), and activated carbon precursors (10). Bataua seeds, although less studied, have shown relevant contents of dietary fiber, phenolic compounds, and structural polysaccharides (11–13).

Despite these antecedents, no study has systematically compared the physicochemical composition and valorization potential of açaí berry and bataua seeds obtained from Bolivian production contexts. Such a comparison is relevant both scientifically—given the differences in botanical classification and structural biology of the two species—and practically, as it can inform integrated waste management strategies in communities that process both fruits. The present work addresses this gap by characterizing seeds of *E. precatoria* from the community of Villa Florida (Pando) and seeds of *O. bataua* from the community of Canadá (Pando), applying the same analytical framework, and comparing results under a multi-criteria valorization approach.

The specific objectives were: (1) to determine the proximate composition and structural fraction composition of both seed types; (2) to assess their antioxidant properties and phenolic content; (3) to compare valorization potential using a multi-criteria scoring matrix; and (4) to evaluate alkaline extraction conditions for cellulose recovery and antioxidant extraction efficiency.

## 2 Materials and methods

### 2.1 Botanical description

Known as “asai” in Bolivia, *Euterpe precatoria* palm trees are monocaules; that is, they develop a single unarmed stem that can reach 25 meters in height and up to 25 cm in diameter. The stem has a light gray color with annular scars left by the fallen leaf sheaths. The globose fruits that, when ripe, have a black or black-violet color with a seed inside. Two varieties have been differentiated in Bolivia: *E. precatoria* var. *precatoria*, which grows in parts of the Amazon with populations growing in sympatry with *E. oleracea*, and *E. precaroria* var. *longivaginata*, which grows on mountain slopes (14–16, 52).

Known as “majo” in Bolivia, *Oenocarpus bataua* palm trees are also monocaules with a solitary trunk up to 30 cm in diameter, which has very spaced internubs and with a total size of 25–30 m in height. Each individual bear between 2 and 4 infrafoliar inflorescences with 200 branched raquillas in a hypuriform order. The fruits are ovoid, black with a glossy and accepting epicarp, blackish purple in color and containing a seed (14, 17).

### 2.2 Plant material and sample preparation

Açaí berry seeds (*E. precatoria*) were obtained from the community of Villa Florida, Provincia Federico Román, department of Pando (coordinates: 12.1986° S, 68.6351° W). Seeds were separated from pulp extraction residues, washed, pre-dried, and ground to a homogeneous flour. Bataua seeds (*O. bataua*) were collected from the community of Canadá, municipality of El Sena, department of Pando, in July 2025. After manual pulp removal, seeds were air-dried at ambient temperature for 48 h, followed by controlled drying at 50 °C for 24 h in a forced-air oven (Quimis Q317M-43). Both materials were milled to 1–2 mm particle size and stored in hermetic dark bags until analysis.

### 2.3 Physicochemical characterization

All analyses were performed in triplicate (*n* = 3) unless otherwise stated, and results are expressed as mean ± standard deviation. Moisture content was determined gravimetrically at 105 °C following Bolivian Standard NB-712 (açaí berry) and AOAC 925.09 (bataua). Ash content was determined by calcination at 550–600 °C following NB-312030 (açaí) and AOAC 942.05 (bataua). Crude lipids were determined by Soxhlet extraction with petroleum ether following NB-312027 (açaí berry) and AOAC 920.39 (bataua). Crude protein was determined by the Kjeldahl method (nitrogen × 6.25) following ISO 5983-2:2009 (açaí berry) and NB 329032:2008/IBNORCA (bataua).

Structural fractions (cellulose, hemicellulose, lignin) were determined by sequential detergent fiber analysis. Neutral detergent fiber (NDF) was quantified with amylase treatment following ISO 16472:2006; acid detergent fiber (ADF) was determined according to ISO 13906:2008; acid detergent lignin (ADL) was obtained by treating the ADF residue with 72% H_2_SO_4_. Hemicellulose was calculated as NDF − ADF; cellulose as ADF − ADL; and lignin as ADL. Total dietary fiber (TDF) was determined by the enzymatic-gravimetric method AOAC 985.29 using sequential *α*-amylase, protease, and amyloglucosidase digestion followed by ethanol precipitation and gravimetric quantification of the residue corrected for protein and ash.

Total lignin in açaí seeds was additionally determined by the acid hydrolysis method ISO 21436:2020, providing both acid-insoluble lignin (AIL) and acid-soluble lignin (ASL) fractions quantified by UV spectrophotometry at 205 nm.

### 2.4 Antioxidant activity and total phenolic content

For açaí berry seeds, antioxidant capacity was evaluated by the DPPH (2,2-diphenyl-1-picrylhydrazyl) radical scavenging assay following López-Mejía et al. (18). Extracts were prepared by ultrasound-assisted extraction (UAE, ultrasonic frequency 40 kHz, ultrasonic power 120 W) in a hydroalcoholic solvent (53) at 35 °C for 20 min; a 3 × 2 factorial design (ethanol concentration: 50, 70, 90% v/v; temperature: 35, 60 °C) was applied to optimize extraction. Results were expressed as IC_50_ (mg/mL). For bataua seeds, phenolic compounds were extracted by maceration with 70% ethanol (pH 2.0) at a 1:10 solid-to-solvent ratio for 2 h at room temperature, followed by rotary evaporation at 50 °C. Total phenolic content (TPC) was quantified by the Folin–Ciocalteu method (19) at 760 nm using gallic acid as standard, expressed as mg gallic acid equivalents per 100 g (mg GAE/100 g). Antioxidant capacity was determined by DPPH at 517 nm and expressed as TEAC (μmol Trolox equivalents per g fresh weight).

### 2.5 Valorization matrix

A multi-criteria scoring matrix was used independently for each species to rank the valorization potential of the main seed fractions. For açaí berry seeds, four criteria were scored 1–5: added value potential, availability/abundance, local technical feasibility, and environmental sustainability [maximum 20 points; (20)]. For bataua seeds, five criteria were scored with differential weights: market demand (max 7), content percentage (max 5), extraction feasibility (max 4), application versatility (max 3), and market price (max 2), totaling a maximum of 20 points (21). Every matrix was conceived as an intra-species valorization tool, and not as an inter-species comparison purpose. Therefore, the purpose of this matrix is to independently prioritize the fractions that could be valued in each species.

### 2.6 Cellulose extraction and FTIR and optical characterization

Alkaline pretreatment was applied to both seed types to isolate cellulose (46). For açaí seeds, a single treatment with 7% NaOH at 75 °C (60 min) was compared with integrated valorization, where antioxidant extraction (optimal treatment: 70% EtOH, 60 °C, UAE) preceded cellulose extraction from the residue. For bataua seeds, a 2 × 2 factorial design was applied: NaOH concentrations of 7 and 10% at temperatures of 75 °C and 110 °C (60 min). In all cases, the Browning holocellulose method (NaClO_2_/acetic acid, 70–80 °C) and the Kennedy cellulose isolation method (24% KOH, 15 h, ambient temperature) were applied sequentially. Cellulose yield was calculated gravimetrically. FTIR spectroscopy was performed using a Shimadzu IR Prestige-21 spectrometer (resolution 0.5 cm^−1^) to evaluate structural changes; cellulose pellets were prepared with KBr at a ratio of 1:100; 20 runs were recorded for each IR spectrum. Statistical analysis was conducted by two-way ANOVA followed by Tukey’s test (*α* = 0.05) using Microsoft Excel. Crystallinity of obtained cellulose was evidenced by observations under polarized light microscope, anisotropic crystals have the property of birefringence which allows them to polarize elliptically polarized light, therefore crystalline regions allow the passage of light under crossed polarizer analyzer conditions. A polarized light microscope (Ernst Leitz GmbH, Wetzlar, Germany) equipped with a halogen light source was used. Observations were carried out using a 10 × objective lens and 6 × ocular lenses (total magnification 60×). The microscope was operated in both plane-polarized light (polarizer only) and cross-polarized light (polarizer and analyzer crossed). Cellulose granules were observed as dry, dispersed powder without mounting medium. The particle size of the granules ranged from 0.5 to 1.5 mm.

## 3 Results and discussion

### 3.1 Proximate and structural composition

Table 1 presents the comparative physicochemical characterization of açaí and bataua seeds. Both species exhibited a markedly lignocellulosic matrix, as evidenced by high TDF values (91.74 ± 0.60% and 84.79 ± 0.98% for açaí and bataua, respectively). These values are consistent with reports in the literature for Arecaceae seeds, where fiber constitutes the dominant fraction (8, 11).

**Table 1:** Comparative physicochemical characterization of açaí berry (*E. precatoria*) and bataua (*O. bataua*) seeds (mean ± SD; *n* = 3).

Parameter	*E. precatoria*	*O. bataua*	Units/Notes
Moisture (whole seed)	32.07 ± 0.91	10.16 ± 0.04	% Fresh basis
Moisture (ground seed)	12.46 ± 0.06	11.09 ± 0.13	% Fresh basis
Ash	0.87 ± 0.19	1.13 ± 0.09	% Dry basis
Crude lipids	2.81 ± 0.17	1.30 ± 0.02	% Dry basis
Crude protein	5.34 ± 0.23	2.00 ± 0.04	% Dry basis
Total dietary fiber (TDF)	91.74 ± 0.60	84.79 ± 0.98	% Dry basis
Cellulose	51.44 ± 0.56	32.58 ± 0.15	% Dry basis (ADF–ADL)
Hemicellulose	18.22 ± 0.20	31.89 ± 0.59	% Dry basis (NDF–ADF)
Lignin (ADL)	20.94 ± 0.73	19.93 ± 0.40	% Dry basis
Antioxidant capacity (DPPH)	IC_50_ = 4.56 ± 0.11 μg/mL TEAC = 4,975.68 ± 56.21 μmol TE/g	IC_50_ = 11.30 ± 0.09 μg/mL TEAC = 1,567.10 ± 16.10 μmol TE/g	
Total phenolic content	3,565.95 ± 21.78 mg GAE/100 g	797.25 mg GAE/100 g	

Cellulose was the dominant structural polysaccharide in açaí seeds (51.44 ± 0.56%), a value higher than the 32–45% range reported by Barros et al. (7) for *E. precatoria*, likely reflecting methodological differences in hemicellulose and lignin removal efficiency. Other physicochemical analyses of “acai seeds” of *E. precatoria* indicate that the dominant cellulose can represent between 34 and 53% of the dry material, with variability linked to region and fruit management (4). A value of 51.44% falls within this upper range and indicates a relatively high degree of cellulose purity in the seed fraction, with relatively low proportions of hemicelluloses, lignin, and extractives. Bataua seeds exhibited a more balanced structural profile: cellulose 32.58 ± 0.15%, hemicellulose 31.89 ± 0.59%, and lignin 19.93 ± 0.40%. These values are coherent with reports for *O. bataua* by Cruz et al. (11) and Mafra et al. (12) and place this species within the typical range of Amazonian palm seeds [cellulose 20–45%, hemicellulose 20–40%, lignin 15–35%; (22)]. The higher hemicellulose-to-cellulose ratio in bataua seeds compared with açaí seeds (∼1:1 vs. ∼0.35:1) implies different reactivity under alkaline pretreatment and may influence selectivity during fractionation.

Moisture content differed substantially between whole and ground seeds for açaí (32.07 ± 0.91% and 12.46 ± 0.06%, respectively), reflecting the dense lignocellulosic structure of the intact seed and rapid surface equilibration upon grinding, a behavior also reported for Brazil nut and açaí seeds (23). Ash content was low in both species (0.87 ± 0.19% açaí; 1.13 ± 0.09% bataua), consistent with Barros et al. (7) and Costa-Singh (24), and indicative of a predominantly organic, low-mineral matrix.

Crude lipid content was low in both species (2.81 ± 0.17% açaí; 1.30 ± 0.02% bataua), confirming that the lipid fraction is concentrated in the mesocarp and not in the seed (8, 25). Crude protein was also low (5.34 ± 0.23% açaí; 2.00 ± 0.04% bataua), consistent with the structural function of these seeds and with prior reports (24, 26). The lower protein content in bataua seeds may reflect greater fiber-to-protein ratios in this species.

### 3.2 Antioxidant capacity and phenolic compounds

The antioxidant profiles of both seed types were characterized by distinct methodologies and units yet consistently revealed biologically relevant activities. For açaí berry seeds, the DPPH assay yielded an IC_50_ of 4.56 ± 0.11 μg/mL for the hydroalcoholic extract optimized at 70% ethanol and 60 °C. This value exceeds the antioxidant potency of the intact *E. precatoria* fruit [IC_50_ = 1,350 ± 90 μg/mL reported by (27)], which is expected given the dilution of phenolics from the pulp, but compares favorably with Previtalli-Silva et al. (9) who reported an IC_50_ of 6.03 μg/mL for the seed of *E. oleracea*, confirming that this fraction presents relevant antioxidant activity; in terms of Trolox equivalent a value 4,975.68 ± 56.21 μmol TE/g was obtained for açaí berry seeds. For bataua seeds, TPC reached 797.25 mg GAE/100 g, close to values reported by Méndez-Durazno et al. (13) (758.25 ± 3.22 mg GAE/100 g) for *O. bataua* from Ecuador, and antioxidant capacity was 1,567.10 ± 16.10 μmol TE/g (TEAC), within the range reported for Peruvian bataua by Vargas-Arana et al. (28).

Concerning the total phenolic content, acai seeds have a higher concentration (3,565.95 ± 21.78 mg GAE/100 g,) compared to bataua seeds (797.25 mg GAE/100 g). Açaí seeds are known to contain procyanidins, catechin, epicatechin, ferulic acid, and anthocyanins partially retained in the seed matrix after depulping (9, 29, 48). Melo et al. (8) reported a content of 6,458 mg GAE/100 g for *Euterpe oleracea* on a dry basis, using ethanol and water at 25 °C. The dense, fibrous structure of the seed can encapsulate polyphenols within the polysaccharide matrix, making extraction efficiency strongly dependent on particle size reduction, solvent polarity, and temperature. Melo et al. (8) demonstrated using Response Surface Methodology that an optimal ethanol concentration of ∼57% maximized phenolic extraction from *E. oleracea* seeds, consistent with the results of the present work for *E. precatoria*, where the 70% ethanol/60 °C treatment yielded the best IC_50_ (2.10 μg/mL; the lowest value indicating highest antioxidant power). For bataua seeds, the Folin–Ciocalteu method with 70% ethanol extraction revealed high polyphenol accumulation, which may include hydroxybenzoic and hydroxycinnamic acids, flavonoids, and condensed tannins documented in the *Oenocarpus* genus (30, 31).

The antioxidant properties of both seed types support their potential application as natural antioxidant ingredients in food, nutraceutical, cosmetic, and pharmaceutical industries, where demand for plant-based bioactive compounds has grown substantially in recent years (32, 33). Stability of phenolic compounds under processing conditions—particularly the thermal sensitivity of anthocyanins and the pH dependence of their chromophore structure (34)—must be considered in scaling up extraction protocols.

### 3.3 Multi-criteria valorization assessment

Table 2 summarizes the valorization scores obtained for each fraction in both species. Despite differences in scoring methodology and maximum points, the results are consistent in identifying cellulose as the top-priority fraction based on abundance and industrial versatility, while antioxidants/phenolics rank very highly due to their elevated market value and ease of extraction.

**Table 2:** Valorization matrix for açaí berry (*E. precatoria*) and bataua (*O. bataua*) seed fractions.

Fraction	*E. precatoria* score*	*O. bataua* score*	Added value	Availability	Priority rank
Cellulose	16/20	19/20	High	High	1st (bataua)/2nd (açaí)
Antioxidants/Phenolics	17/20	16/20	Very high	Moderate	1st (açaí)/2nd (bataua)
Hemicellulose	13/20	14/20	Moderate	High	3rd
Lignin	13/20	11/20	Moderate	Moderate	4th
Lipids	9/20	8/20	Low	Low	5th
Protein	10/20	9/20	Low–Moderate	Low	5th–6th

*Scores based on Iacovidou and Voulvoulis (20) criteria for açaí berry and Peters and Peters (21) criteria for bataua.

For açaí seeds, antioxidants received the highest score (17/20), reflecting their elevated market value and the demonstrated experimental activity, even though their extraction precedes cellulose recovery in the proposed integrated process. Cellulose scored second (16/20) due to its outstanding abundance (51.44%) and its applications in biomaterials, pharmaceutical excipients, and biocomposites. For bataua seeds, cellulose topped the ranking (19/20) driven by its high content, consolidated market demand, and process feasibility, while phenolic compounds ranked second (16/20) given their superior market value and extraction simplicity. In both species, hemicellulose, lignin, lipids, and proteins received lower scores, primarily due to lower abundance (lipids, proteins), greater extraction complexity (lignin), or less consolidated markets (hemicellulose) at the current stage of technology readiness (47).

These results suggest that an integrated valorization cascade—first extracting antioxidant-rich fractions with hydroalcoholic solvents, then recovering cellulose from the remaining solid residue—represents an economically and environmentally sound strategy for both seed types. This approach is consistent with biorefinery principles and with the circular economy mandate of maximizing resource utility and minimizing waste (35).

### 3.4 Cellulose extraction and FTIR characterization

Table 3 summarizes cellulose yields obtained under different alkaline conditions. For bataua seeds, a 2 × 2 factorial ANOVA confirmed significant effects of NaOH concentration (*F* = 488.57, *p* < 0.001), temperature (*F* = 1132.06, p < 0.001), and their interaction (*F* = 332.09, p < 0.001) on cellulose yield. Temperature was the dominant factor. Tukey post-hoc analysis grouped the 7% NaOH–110 °C and 10% NaOH–110 °C treatments in the same statistical group (A), with no significant difference between them (29.33 ± 0.01% and 28.97 ± 0.15%), both significantly higher than the 7% NaOH–75 °C treatment (24.06 ± 0.29%; group C).

**Table 3:** Cellulose extraction yields and FTIR assessment under different alkaline pretreatment conditions for both palm species (mean ± SD; *n* = 3).

Treatment	*E. precatoria* yield (%)	*O. bataua* yield (%)	FTIR lignin removal	FTIR cellulose integrity
7% NaOH – 75 °C	31.8 ± 2.8*	24.06 ± 0.29	Effective	Preserved
10% NaOH – 75 °C	—	27.66 ± 0.37	Effective	Preserved
7% NaOH – 110 °C	—	28.97 ± 0.15	High	Preserved
10% NaOH – 110 °C	—	29.33 ± 0.01	High	Preserved
70% EtOH – 60 °C (antioxidant extraction, then NaOH 7%)^†^	21.2 ± 1.9	N.A.	Medium	Medium crystallinity

*Yield expressed relative to antioxidant-extraction residue dry weight. N.A.: not applicable. †EtOH means Ethanol.

To substantiate the assertion that temperature is the dominant factor controlling cellulose yield, effect-size metrics and confidence intervals were computed from the complete ANOVA summary (see Table 4). Eta squared (*η*^2^), which expresses the proportion of total variance attributable to each source of variation, indicated that temperature accounted for 57.74% of the total variance in cellulose yield, NaOH concentration for 24.92%, and their interaction for 16.94%, leaving only 0.41% unexplained (*R*^2^ = 0.996). Partial eta squared (*η*^2^p), an alternative metric commonly reported in factorial designs, yielded values of 0.993 for temperature, 0.984 for NaOH concentration, and 0.976 for the interaction—all well above (54) threshold for a “large” effect (*η*^2^p ≥ 0.14). Omega squared (ω^2^), a less biased estimator that corrects for sampling error, produced nearly identical proportions: 57.66% for temperature, 24.85% for NaOH concentration, and 16.88% for the interaction, confirming the stability of the variance partition across estimation methods.

**Table 4:** Effect-size estimates and 95% confidence intervals for cellulose yield under alkaline pretreatment of bataua (*Oenocarpus bataua*) seeds.

Source of variation	SS	df	MS	*F*	*p*	*η* ^2^	*η*^2^p	*ω* ^2^	*f* ^2^
Temperature (°C)	30.349	1	303.49	1132.06	6.65 × 10^−10^	0.5774	0.9930	0.5766	141.49
NaOH concentration (%)	13.098	1	130.98	488.57	1.85 × 10^−8^	0.2492	0.9839	0.2485	61.06
Temperature × NaOH	8.903	1	89.03	332.09	8.45 × 10^−8^	0.1694	0.9765	0.1688	41.50
Error	0.215	8	0.027	—	—	0.0041	—	—	—
Total	52.564	11	—	—	—	1.0000	—	—	—

Treatment	Mean ± SD	95% CI
7% NaOH, 75 °C	24.06 ± 0.29	[23.84, 24.28]
10% NaOH, 75 °C	27.66 ± 0.37	[27.44, 27.88]
7% NaOH, 110 °C	28.97 ± 0.15	[28.75, 29.19]
10% NaOH, 110 °C	29.33 ± 0.01	[29.11, 29.55]

SS, sum of squares; MS, mean square; *η*^2^, eta squared (proportion of total variance); *η*^2^p, partial eta squared; *ω*^2^, omega squared; *f*^2^, Cohen’s f-squared; CI, confidence interval computed from the pooled ANOVA error term (MS_error = 0.0268, SEM = 0.0945, t_0.025, 8 = 2.306).

To aid process optimization, 95% confidence intervals (CI) were computed for each treatment mean using the pooled error term from the ANOVA (MS_error = 0.0268, SEM = 0.0945, t_0.025, 8 = 2.306). The resulting intervals are shown in Table 4. None of the 75 °C intervals overlapped with any 110 °C interval, confirming that the temperature shift from 75 °C to 110 °C produced a practically meaningful increase in yield irrespective of NaOH concentration. Conversely, the intervals for the two 110 °C treatments overlapped (29.11–29.19%), indicating that increasing NaOH from 7 to 10% at 110 °C did not yield a statistically or practically significant gain. This pattern is consistent with the variance-partition analysis: once temperature is elevated, NaOH concentration becomes a marginal factor.

Collectively, these metrics—*η*^2^, *ω*^2^, *f*^2^, and non-overlapping confidence intervals—provide convergent evidence that temperature is the principal driver of cellulose yield in alkaline pretreatment of bataua seeds. From an operational standpoint, this implies that near-optimal yields can be achieved at 110 °C with 7% NaOH, avoiding the higher chemical consumption and potential cellulose degradation associated with 10% NaOH. This interpretation is particularly relevant for small-scale biorefinery operations in Amazonian communities where reagent availability and energy costs are limiting factors.

For açaí berry seeds, cellulose recovery from the antioxidant-extraction residue yielded 21.2 ± 1.9%, a value expressed as mass yield relative the original seed; for the direct extraction of cellulose from the origin the yield increases to 31.8 ± 2.8%, this is consistent with the high cellulose content of the original seed and the efficiency of sequential extraction.

FTIR spectroscopy confirmed effective delignification and hemicellulose removal in both species. The characteristic O–H stretching band (3,300–3,400 cm^−1^) was preserved in all treatments, confirming cellulose hydroxyl group integrity. The C–H stretching band at ∼2,900 cm^−1^ was stable across treatments, indicating no significant degradation of the glucosidic backbone, as discussed by Alemdar and Sain (36) and confirmed here for both species. The disappearance or strong attenuation of the carbonyl band at 1,730 cm^−1^ (acetyl groups in hemicellulose) and the aromatic bands at 1,510–1,600 cm^−1^ (lignin) at higher severity treatments confirmed successful saponification and solubilization of these components (11, 37). The I_1420_/I_895_ ratio, a structural order indicator, increased with treatment severity in both species, consistent with reduced amorphous regions and higher relative crystallinity, as reported for NaOH-treated natural fibers (38, 39).

**Figure 1:**
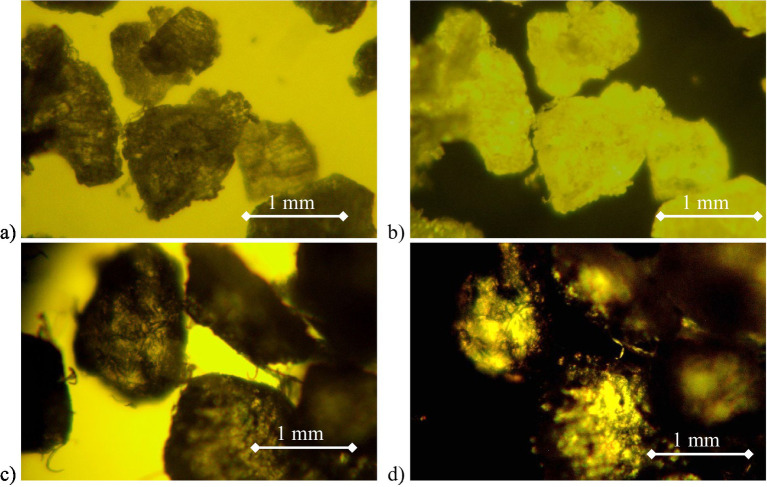
Above: cellulose particles obtained from acai seeds observed with polarized light under: **(a)** parallel polarizer/analyzer and, **(b)** crossed polarizer/analyzer configurations. Below: the same observations for bataua cellulose particles. On the left **(c)** we see that the particles appear dark and on the right **(d)** the particles appear bright thanks to the elliptical polarization of polarized light due to birefringence of cellulose crystalline regions, amorphous regions appear darker under crossed polarizer/analyzer configurations.

As a qualitative evidence of crystallinity in both cellulose extracts, particles of cellulose of acai and bataua were observed under polarized light with crossed polarizer/analyzer, and parallel polarizer/analyzer configurations. As it is shown on Figure 1, under parallel polarizer/analyzer configuration, cellulose particles appear darker than the field, but when observed under crossed polarizer/analyzer configuration, the crystalline portion of the particles appear bright in a dark field, this is due to the birefringence of cellulose crystalline regions. Some regions appear darker due to an amorphous structure of those regions. This a clear evidence of crystallinity of the cellulose particles. These observations will be complemented with XRD studies to obtain a quantitative crystallinity index, in the near future.

Importantly, the 75 °C treatments for bataua seeds already yielded cellulose with adequate structural purity, suggesting that lower-temperature processing may represent a more sustainable option when the balance between quality, energy consumption, and operational cost is considered (40). This finding is particularly relevant for small-scale processing in Amazonian communities with limited energy infrastructure. For açaí berry seeds, the medium crystallinity of the cellulose extracted after antioxidant recovery, confirmed by FTIR, suggests suitability for applications such as microcrystalline cellulose production, biodegradable composites, or cellulose nanocrystal precursors (41).

### 3.5 Comparative analysis and valorization strategies

Despite originating from the same geographic region and sharing a lignocellulosic seed structure, *E. precatoria* and *O. bataua* seeds present complementary compositional profiles that inform distinct but overlapping valorization strategies.

Açaí seeds are characterized by a higher cellulose content and well-documented antioxidant activity driven by procyanidins and other polyphenols partially retained in the seed matrix after depulping (9, 26). The integrated extraction cascade (antioxidants first, cellulose second) allows maximal value recovery from a single residue stream, consistent with the sequential biorefinery concept (6). The high lignin content (∼20%) confers structural resistance that, while requiring mechanical pretreatment (milling) before extraction, also positions the remaining lignocellulosic matrix as a precursor for biochar, activated carbon, and soil amendment applications—uses already demonstrated for açaí seeds (10, 42, 50).

Bataua seeds showed a more balanced structural distribution, with roughly equal proportions of cellulose and hemicellulose, which implies that hemicellulose-derived products (fermentable sugars, xylose, bioplastics) also warrant consideration as secondary valorization routes. The high TPC (797.25 mg GAE/100 g) and TEAC (1567.10 μmol TE/g) confirm substantial phenolic accumulation in the seed, making antioxidant extraction a viable primary step before structural fractionation. The relatively lower protein content compared with açaí seeds (2.00% vs. 5.34%) reduced the need for nitrogen correction in enzymatic analyses and simplifies the extraction process.

From a comparative standpoint, the convergence of both species on cellulose and antioxidants as priority fractions strengthens the case for standardized processing protocols applicable to both residue streams in Pando region. Communities that process both fruits could adopt common equipment—ultrasound baths, alkaline reactors, and vacuum filtration systems—reducing capital investment and enhancing the scalability of valorization operations.

The lignocellulosic fractions of both seeds also position them within the rapidly growing nanocellulose market. Cellulose nanocrystals (CNCs) and cellulose nanofibers (CNFs) derived from non-wood sources have attracted considerable industrial interest due to their mechanical properties, biodegradability, and surface functionality (41). The relatively moderate lignin contents of both species (∼20%) suggest that moderate pretreatment conditions may suffice to produce nanocellulose-grade raw material, pending further characterization by X-ray diffraction, scanning electron microscopy, and degree-of-polymerization analysis.

### 3.6 Sustainability considerations and limitations

Both studies were conducted under the legal framework of Bolivian environmental legislation, including Law 755 on Integral Waste Management, Law 1,333 on the Environment, and Law 300 on Mother Earth and Integral Development for Living Well, which collectively promote the valorization of organic residues through clean technologies (43, 44). The proposed valorization pathways align with these normative imperatives while also responding to the growing global demand for plant-derived bioactive ingredients and bio-based materials.

Several limitations of the present study must be acknowledged. First, both seed characterizations were performed on samples from single communities and single harvest seasons, and natural variability in seed composition due to soil type, rainfall, and fruit maturity is expected (45). Second, integrated valorization cascade proposed for acai is still a conceptual valorization strategy, more studies are needed in order to determine to assess the actual techno-economic viability, however our results show that this is chemically possible. Third, the cost analyses suggest that cellulose production at laboratory scale carries high unit costs relative to commercial cellulose, and economic viability will depend on process intensification and scale-up. Fourth, no toxicological assessment of the extracts was included, which is necessary before food or nutraceutical applications can be validated.

## 4 Conclusion

This study provides a comprehensive comparison of the physicochemical composition and valorization potential of açaí berry (*Euterpe precatoria*) and bataua (*Oenocarpus bataua*) seeds sourced from communities in the Bolivian Amazon (Villa Florida and Canadá, department of Pando). Both species present predominantly lignocellulosic matrices with high total dietary fiber (>84% dry basis), low lipid contents (<3%), and low protein contents (<6%), confirming their structural nature and suitability for fiber-oriented valorization pathways.

Açaí seeds are distinguished by exceptionally high cellulose content (51.44 ± 0.56% dry basis) and significant antioxidant capacity (IC_50_ = 4.56 μg/mL; TEAC = 4,975.68 ± 56.21 μmol TE/g), positioning them as prime candidates for sequential biorefinery approaches. Bataua seeds exhibit a more balanced polysaccharide profile, with comparable cellulose (32.58 ± 0.15%) and hemicellulose (31.89 ± 0.59%) contents, alongside high total phenolic content (797.25 mg GAE/100 g) and moderate antioxidant activity (TEAC = 1,567.10 ± 16.10 μmol TE/g), which supports their inclusion in similar integrated valorization schemes.

The multi-criteria valorization analysis converged on cellulose and antioxidant/phenolic compounds as the top-priority fractions for both species, despite differences in scoring methodologies. This convergence strengthens the case for standardized, community-scale processing protocols applicable to both residue streams in the Pando region. The proposed integrated extraction cascade—antioxidant recovery using hydroalcoholic solvents followed by alkaline delignification and cellulose isolation—maximizes value recovery from a single residue stream, consistent with circular economy and biorefinery principles.

Alkaline pretreatment with NaOH effectively delignified both seed types without significant degradation of the cellulose glucosidic backbone, as confirmed by FTIR spectroscopy. Temperature was the dominant factor controlling cellulose yield in bataua seeds, with 110 °C treatments achieving the highest yields (∼29%), although 75 °C conditions already provided adequate structural purity and may represent a more sustainable option for small-scale operations with limited energy infrastructure. Polarized-light microscopy provided qualitative evidence of cellulose crystallinity in both species, complementing the FTIR structural assessment.

From a sustainability perspective, the valorization of these agroindustrial residues aligns with Bolivian environmental legislation (Laws 755, 1,333, and 300) and contributes to bioeconomy objectives in the Amazon by generating additional income for local communities while reducing organic waste accumulation. However, several limitations must be acknowledged: samples originated from single communities and harvest seasons, natural compositional variability is expected; the integrated cascade remains a conceptual strategy requiring techno-economic validation; laboratory-scale cellulose production carries high unit costs; and toxicological assessment of extracts is necessary prior to food or nutraceutical applications.

Future research should prioritize process scale-up and intensification, quantitative crystallinity determination by X-ray diffraction, nanocellulose production feasibility, phenolic bioavailability studies, and comparative life-cycle analysis of the proposed valorization routes. The complementary compositional profiles of *E. precatoria* and *O. bataua* seeds suggest that communities processing both fruits could adopt common extraction infrastructure, reducing capital investment and enhancing the scalability of circular bioeconomy operations in the Bolivian Amazon.

## Data Availability

The original contributions presented in the study are included in the article/supplementary material, further inquiries can be directed to the corresponding author.
